# Unraveling the therapeutic efficacy of resveratrol in Alzheimer’s disease: an umbrella review of systematic evidence

**DOI:** 10.1186/s12986-024-00792-1

**Published:** 2024-03-19

**Authors:** Ali Azargoonjahromi, Fatemeh Abutalebian

**Affiliations:** 1 Shiraz University of Medical Sciences, Shiraz, Iran; 2 Department of Biotechnology and Medicine, Islamic Azad University of Tehran Central Branch, Tehran, Iran

**Keywords:** Resveratrol, Alzheimer’s, Beta-amyloid plaques, Neurofibrillary tangles, Tau proteins, Neuroprotection, Cognitive function

## Abstract

**Context:**

Resveratrol (RV), a natural compound found in grapes, berries, and peanuts, has been extensively studied for its potential in treating Alzheimer’s disease (AD). RV has shown promise in inhibiting the formation of beta-amyloid plaques (Aβ) and neurofibrillary tangles (NFTs), protecting against neuronal damage and oxidative stress, reducing inflammation, promoting neuroprotection, and improving the function of the blood–brain barrier (BBB). However, conflicting results have been reported, necessitating a comprehensive umbrella review of systematic reviews to provide an unbiased conclusion on the therapeutic effectiveness of RV in AD.

**Objective:**

The objective of this study was to systematically synthesize and evaluate systematic and meta-analysis reviews investigating the role of RV in AD using data from both human and animal studies.

**Data sources and extraction:**

Of the 34 systematic and meta-analysis reviews examining the association between RV and AD that were collected, six were included in this study based on specific selection criteria. To identify pertinent studies, a comprehensive search was conducted in English-language peer-reviewed journals without any restrictions on the publication date until October 15, 2023. The search was carried out across multiple databases, including Embase, MEDLINE (PubMed), Cochrane Library, Web of Science, and Google Scholar, utilizing appropriate terms relevant to the specific research field. The AMSTAR-2 and ROBIS tools were also used to evaluate the quality and risk of bias of the included systematic reviews, respectively. Two researchers independently extracted and analyzed the data, resolving any discrepancies through consensus. Of note, the study adhered to the PRIOR checklist.

**Data analysis:**

This umbrella review presented robust evidence supporting the positive impacts of RV in AD, irrespective of the specific mechanisms involved. It indeed indicated that all six systematic and meta-analysis reviews unanimously concluded that the consumption of RV can be effective in the treatment of AD.

**Conclusion:**

RV exhibits promising potential for benefiting individuals with AD through various mechanisms. It has been observed to enhance cognitive function, reduce Aβ accumulation, provide neuroprotection, protect the BBB, support mitochondrial function, facilitate synaptic plasticity, stabilize tau proteins, mitigate oxidative stress, and reduce neuroinflammation commonly associated with AD.

**Graphical abstract:**

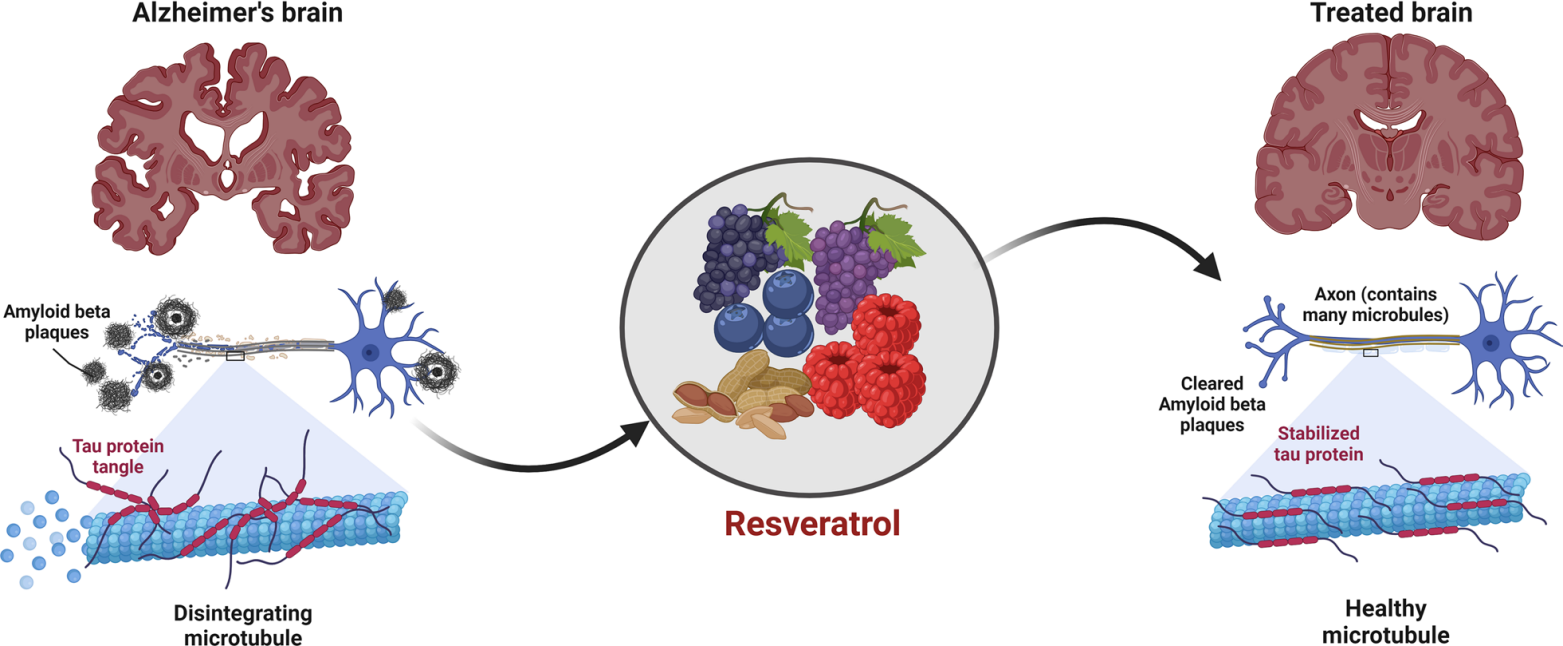

**Supplementary Information:**

The online version contains supplementary material available at 10.1186/s12986-024-00792-1.

## Introduction

Alzheimer’s disease (AD), the primary cause of dementia accounting for 60% to 80% of cases [1–3], is a progressive neurological disorder characterized by the presence of abnormal protein deposits in the brain such as beta-amyloid (Aβ) plaques and neurofibrillary tangles (NFTs) composed of tau protein [4–7]. These deposits disrupt cellular processes, impair communication between neurons, and contribute to the degeneration and death of brain cells [6, 8], resulting in memory loss, cognitive decline, and behavioral changes [9, 10].

All currently approved medications for the treatment of AD were authorized more than a decade ago. The primary drugs used as initial treatment options are acetylcholinesterase (AChE) inhibitors, namely donepezil, rivastigmine, and galantamine. These medications work by blocking the breakdown of acetylcholine, a neurotransmitter that plays a role in memory and cognitive function [12–14]. They are utilized to alleviate cognitive impairment and slow down the progression of dementia in AD patients [15]. Memantine, an NMDA receptor antagonist, has been approved for moderate-to-severe AD [16, 17].

Most importantly, the US FDA has approved aduhelm (aducanumab) and Leqembi (lecanemab-irmb) for treating AD patients (see https://www.fda.gov/drugs/news-events-human-drugs/fdas-decision-approve-new-treatment-alzheimers-disease and https://www.fda.gov/news-events/press-announcements/fda-converts-novel-alzheimers-disease-treatment-traditional-approval), marking them medications to target Aβ in the brain. Despite doubts about their clinical efficacy, these monoclonal antibodies have shown to decline Aβ; as a result, reducing Aβ can reduce the clinical decline of AD [6, 18].

Various substances, including nutrients, are now recognized as effective in early stages of AD, supporting brain function and slowing down AD progression [6, 12, 13, 19, 20]. Resveratrol (RV), also known as 3,4′,5-trihydroxystilbene (chemical formula: C14H12O3), is an epitome of such substances. RV, a polyphenolic phytoalexin found in grapes, berries, and certain plants, has been linked to potential health benefits like neuroprotective and anti-inflammatory properties [26, 27]. RV exists in two forms, -trans and -cis isomers, and notably, previous studies have shown that trans-RV, found in higher concentrations than cis-RV in foods like grapes and wine [29–31], exhibits greater antioxidant and anti-inflammatory properties [32].

The notion that RV could serve as a therapeutic agent to manage the progression of AD has been influential in recent decades, prompting numerous researchers to investigate the potential role of RV in AD [33–36]. For instance, recent research indicates that RV may play a role in anti-amyloidogenic mechanisms, suggesting that natural or synthetic analogues may have therapeutic potential in AD [36]. RV may also potentially impact AD through its antioxidant, silent information regulator-1 (SIRT1)-activating [37], and anti-inflammatory properties [33], as well as its ability to regulate Aβ and tau protein, both of which are involved in the development of AD. RV has been suggested to offer neuroprotection, promote neurogenesis, [38], and mitigate oxidative stress in the brain to control AD [37]. Nevertheless, it is important to note that not all studies have reported promising results regarding the role of RV in AD. In fact, the findings from various studies are controversial, and some studies have not specifically reported a positive impact of RV on cognitive and memory performance, which are characteristics associated with AD [39, 40].

Given the foregoing debate regarding the role of RV in AD, numerous systematic reviews have been conducted. However, no comprehensive review has yet synthesized the relevant evidence, leaving the overall benefits of RV use for AD unclear. This means that no study has undertaken as a comprehensive review that incorporates a broad spectrum of relevant studies, diverse study designs, meticulous evidence analysis, inclusion of studies conducted on diverse populations, and a thorough assessment of the impact of RV on AD. Indeed, existing systematic reviews investigating the role of RV in AD exhibit limitations stemming from diverse study designs, participant characteristics, dosage regimens, treatment durations, and outcome measures. The variability in RV dosages and formulations, influenced by factors such as food intake and metabolism, introduces the possibility of inconsistent findings across studies. Additionally, some studies fail to adequately account for potential confounding factors, including genetic predisposition, coexisting medical conditions, and medication use, which can potentially modify the effects of RV. Therefore, our objective is to evaluate the strength and credibility of the evidence derived from systematic and meta-analysis reviews on RV intake in AD by conducting an umbrella review to reach a convergent conclusion.

Umbrella reviews offer a comprehensive synthesis of evidence by integrating findings from multiple systematic and meta-analysis reviews. They assess quality, identify discrepancies, and evaluate evidence strength, providing time and resource efficiency, supporting decision-making, identifying research gaps, and optimizing resource utilization. Indeed, umbrella reviews consolidate data from multiple systematic reviews on a specific topic, providing a comprehensive overview of the research domain. They have the ability to identify patterns, contradictions, or areas that may require further investigation, which individual reviews might overlook [41, 42].

This umbrella review will synthesize and analyze the available evidence, considering the diverse study designs, participant characteristics, dosage variations, treatment durations, and outcome measures reported in existing systematic reviews. By doing so, it aims to provide a more comprehensive understanding of the relationship between RV and AD, while also accounting for potential confounding factors. This umbrella review will analyze six systematic and meta-analysis reviews that investigated the relationship between RV and AD based on predefined selection criteria.

## Methods and materials

This present umbrella review was conducted in accordance with the PRIOR (Preferred Reporting Items for Overviews of Reviews) guidelines [43]. The PRIOR checklist is presented in Additional file 1: Appendix 1.

### Eligibility criteria

We included systematic and meta-analysis reviews that assessed the impact of RV on AD in both human and animal studies. We have included both animal and human studies in this review because it is crucial for the evaluation of interventions [44–47]. Animal studies provide valuable insights into mechanisms, efficacy, and safety, serving as a bridge between basic research and clinical applications [48, 49]. They offer predictive value by exploring intervention effects before testing them in humans [49]. Animal studies also help identify potential harms and safety concerns [50]. By understanding the underlying biological mechanisms through animal studies, researchers can develop targeted treatments for humans. Furthermore, including animal studies respects ethical boundaries and allows exploration of interventions that may not be feasible or ethical in humans [47, 51–53]. Thus, integrating animal and human studies in an umbrella review provides a more robust evidence base, supporting informed decision-making in both preclinical and clinical research.

We defined systematic reviews as peer-reviewed studies that follow a specific methodology, including having a clearly reported research question, conducting a systematic search of at least two databases, and performing systematic data synthesis. Importantly, those systematic reviews that followed the PRISMA guidelines were included. These reviews aim to provide a comprehensive and unbiased summary of existing evidence on a specific topic—the role of RV in AD. Additionally, the eligibility criteria used in other overviews of systematic reviews should be replicated to ensure consistency across different reviews. Certain types of reviews were excluded from the definition of systematic reviews: Reviews with only one author were excluded because systematic reviews typically involve a team of researchers working together to minimize bias and enhance reliability; reviews that searched only one database were also excluded, as this can lead to incomplete coverage of relevant studies.

### Information sources and search strategy

We conducted an extensive search across multiple databases, including Embase, MEDLINE (PubMed), Cochrane Library, Web of Science, Epistemonikos, and Google Scholar, with the aim of finding relevant research. Studies must be written in English and published in peer-reviewed journals. The search was not restricted by the publication start date and covered until October 15, 2023. We utilized specific terms such as “resveratrol,” “wine,” “SIRT1 activator,” “3,5,4′-trihydroxy-trans-stilbene,” “cis- and trans-resveratrol,” “peanuts,” and “resveratrol supplement”. These terms were combined with keywords associated with “Alzheimer's disease,” “neurodegenerative conditions,” “cognitive function,” “beta-amyloid,” “neurofibrillary tangles,” “tau protein,” “systematic review,” and “meta-analysis”.

To clarify, we conducted thorough searches in mentioned databases by combining different terms. For instance, we explored “resveratrol” combined with “Alzheimer’s disease” in each database, then “resveratrol” combined with “neurodegenerative conditions”, and so forth. We experimented with diverse search methods, using quotation marks, Boolean operators and incorporating these terms into sentences. It is important to note that our searches were not limited to specific words; instead, these words were integral components of sentences crafted for each database. Additional file 2: Appendix 2 shows details of the search strategy used in the present umbrella review.

In addition to the database search, we conducted a thorough review of the reference lists in relevant review papers and papers that met our study entry criteria. This supplementary step enabled us to discover further published research by examining the references cited within those papers.

### Selection process

We sorted the included reviews based on the population and intervention comparisons (known as PICOs) while ensuring adherence to the PRISMA guidelines [54]. In cases where multiple reviews addressed the same comparison for the same population, we prioritized the review with the most recent search date and completeness of the search, as well as the highest quality. To assess overlap, the first author (A.A.) extracted this information from the reviews, and the second author (F.A.) cross-verified the data. Additionally, we evaluated the methodological quality of the included reviews using a checklist specifically designed for systematic reviews called AMSTAR-2 (A MeaSurement Tool to Assess systematic Reviews) [55]. Both authors (A.A. and F.A.) independently assessed each publication and reached a consensus on the quality through discussions. Any studies that did not meet the criteria outlined in AMSTAR-2 were excluded from the analysis. The final decision on which reviews to include was made through agreement between two of the authors (A.A. and F.A.).

### Data collection process

Two of the authors involved in the overview independently extracted data from each systematic review using an electronic form that was created and tested beforehand. Any disagreements that arose were resolved through consensus among the authors. In the event that inconsistent data were identified across the systematic reviews, our plan was to extract data from all the included reviews and address the discrepancies by contacting the authors of those reviews, retrieving primary studies from the included reviews, and searching relevant trial registries. We intended to discuss any potential discrepancies in the Results section of this umbrella review; however, it is worth noting that no inconsistent data were identified during the extraction process.

### Data items, synthesis methods, risk of bias assessment

Research has been conducted on the connection between RV consumption and AD in both animal and human systematic reviews. Of these six systematic reviews [56–61], four were systematic reviews involving human subjects [56, 58, 60, 61], while two involved animal subjects [57, 59]. We excluded certain studies from the analysis for various reasons. Firstly, studies that assessed AD but focused on components other than RV due to lack of sufficient data were excluded. The presence of a confounder in this situation raises the question of whether RV has influenced AD symptoms or if other components have played a role. Additionally, studies that evaluated only SIRT1 or polyphenols and nutrients without any administration of RV were also excluded. The reason for this is that SIRT1 can be influenced by various other conditions and components [62–64], leading to potential bias. Furthermore, studies that examined wine but did not measure RV within them were excluded as well. This is because wine contains numerous other components that have been demonstrated to have similar effects as RV can [65].

To conduct the screening process, two authors independently reviewed the titles and abstracts of relevant publications. For doing so, we used both EPPI-Reviewer (version: 6.15.0.2) and Microsoft excel 2021; This enabled us to effectively remove duplicate records and perform subsequent screening. Any discrepancies or conflicts were carefully reviewed and reanalyzed in order to reach a resolution. Publications that raised doubts about their context or eligibility were included in the list for full-text screening.

In determining the risk of bias of a systematic review with meta-analysis, we relied on the Risk of Bias in Systematic Reviews (ROBIS) tool [66]. Specifically, we considered a systematic review with meta-analysis to be of high quality if it demonstrated low risk of bias in the first three domains of the ROBIS tool. These domains encompassed the specification of study eligibility (domain 1), the methods used to identify and select studies (domain 2), and the methods used to collect data and appraise studies (domain 3). In order to assess studies that required additional scrutiny, we employed the Cochrane “Risk of Bias” tool [67]. This tool provided a framework for a comprehensive evaluation of the potential biases present in those studies.

## Results

**Fig. 1:**
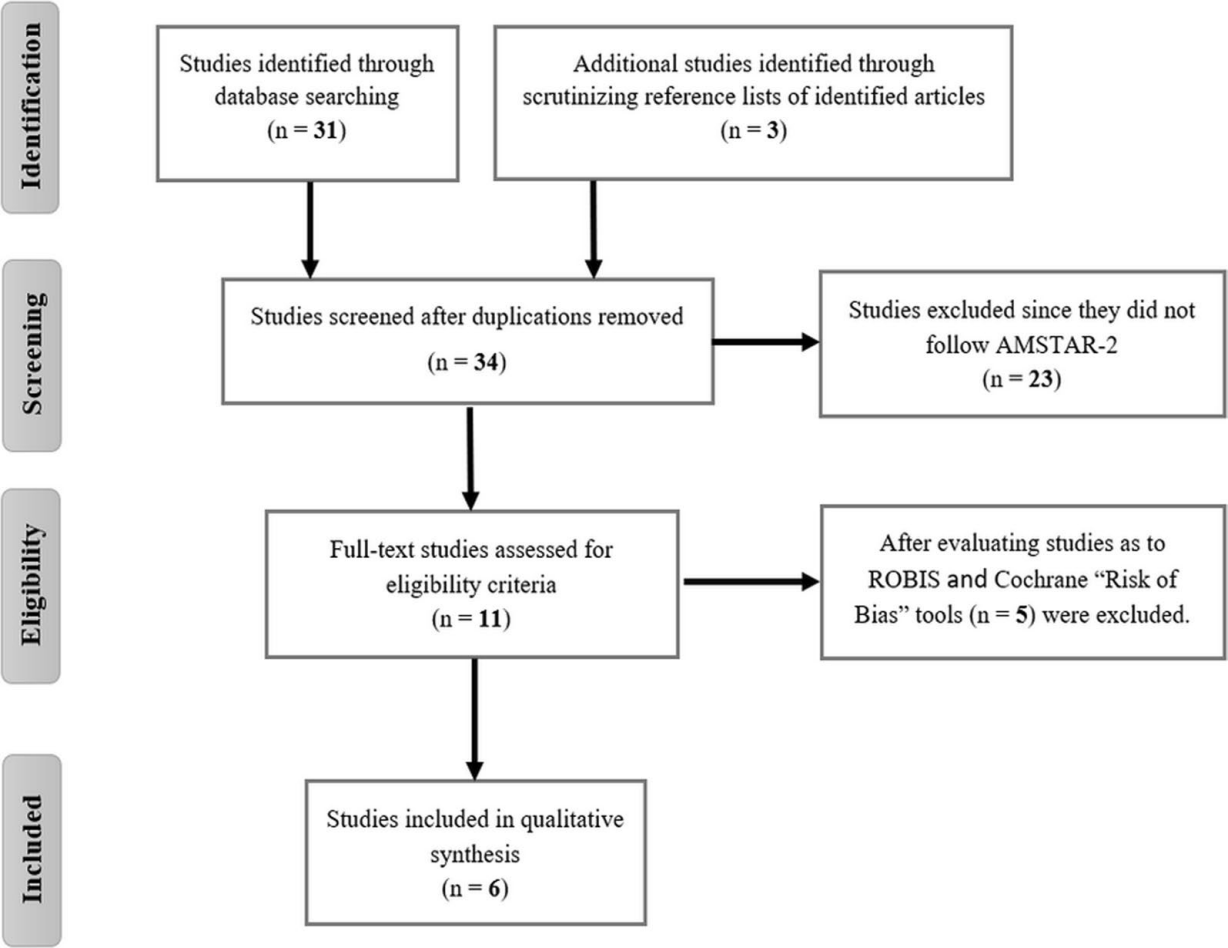
The flowchart of the selection of pertinent studies

A total of 31 studies were collected through multiple databases, and three articles were found by carefully examining the reference lists of publications that had already been identified. Out of the total of 34 systematic reviews, we carefully selected only 6 for systematic evaluation. The reason for this selection was that some of the reviews failed to meet the inclusion criteria as determined by the AMSTAR-2 and ROBIS tools. Additionally, certain reviews did not adhere to the recommended PRISMA guidelines. Moreover, several reviews were found to be rife with confounding factors. For instance, these reviews examined the simultaneous administration of other components alongside RV, making it challenging to ascertain the precise impact of RV alone. Furthermore, some studies neglected to consider comorbid conditions when analyzing the effects of RV on AD patients. This introduced a potential bias, as the AD patients in question were also afflicted with other diseases or disorders such as stroke, depression, diabetes, and cardiovascular diseases, among others. Given this scenario, there are additional factors at play that can influence the impact of RV, thereby preventing us from reaching a definitive conclusion. Thus, six studies [56–61] were evaluated in this umbrella review. Figure 1 shows a flowchart of the selection of pertinent studies.

Out of these six studies [56–61], four were systematic reviews involving human subjects [56, 58, 60, 61], while two involved animal subjects’ systematic reviews [57, 59]. The methodological details of studies have been summarized in Table 1, which followed PICOS (Population, Intervention, Comparison, Outcomes, and Study) criteria.

**Table 1:** The methodological details of analyzed studies

Author, date	Design study	Databases search	Eligibility criteria	Search period	Population	Intervention/ comparison	Outcome	Refs
Buglio et al., 2022	Human systematic review	MEDLINE-PubMed, Cochrane, and EMBASE	Randomized Clinical Trials	Over 10 years up to May, 2021	151 patients with AD (83 women and 68 men)/aged between 49 and 80 years old	Original studies involving patients with AD who were treated with RV	RV brings beneficial effects to patients with AD	[56]
Tosatti et al., 2022	Human systematic review	MEDLINE, CENTRAL, Embase, CINAHL, Web of Science, and Scopus	Randomized, placebo-controlled, clinical trials	Up to August, 2021	241 patients with AD, with 117 patients allocated to the placebo group with a mean age of 75.4 ± 5.2 years, and 124 patients in the intervention group	Original studies involving patients with AD who were treated with RV	RV is effective for cognitive and functional decline in AD patients, when compared with a placebo group	[60]
Kocatürk et al. 2022	Human systematic review	PubMed, web of Science, Korean Journal Database, Russian Science, Citation index, ScieLO Citation index, Cochrane Library and Scopus	Not restricted	Up to 16 April, 2021	186 patients with AD	Original studies involving patients with AD who were treated with RV	RV improvs cognitive, functional prognosis and quality of life in AD patients	[58]
Xu Lou et al., 2023	Human systematic review	PubMed, Web of Science, Scopus, and Cochrane Library	Randomized, placebo-controlled, clinical trials, and systematic studies	2018 to 2022	159 patients with AD (77 patients received RV/ 75 received placebo)	Original studies involving patients with AD who were treated with RV	RV acts as an AD antagonist, fulfilling neuroprotective functions, improving inflammation levels, and promoting cognitive functions	[61]
Chen et al., 2019	Animal systematic review and meta-analysis	Google Scholar, PubMed, Web of Science	Not restricted	Up to August, 2019	19 studies describing the efficacy of RV in rodent AD models by electronic and manual retrieval	Laboratory animals of any breed, age, sex, or strain/ control and resveratrol administration groups based on route of administration, duration of treatment and dosage	RV can be used as anti-AD in the pre-clinical studies, and RV has neuroprotective effects in AD models	[57]
Komorowska et al., 2020	Animal systematic review	PubMed	Not restricted	Over 10 years up to February, 2020	N/A	Animal models of AD received RV	RV can be effectively used in the prevention of AD	[59]

### Human systematic reviews

According to a systematic review of three randomized clinical trials, [56] RV and its metabolites have the potential to traverse the blood–brain barrier (BBB), potentially influencing cognitive function and brain metabolism. They discovered some exciting results, but there was no significant loss observed in a particular segment they were investigating. They also examined various biomarkers and found that while some of their trajectories or patterns had changed, there were no apparent treatment effects on specific factors related to AD, such as plasma plasma Aβ42, CSF Aβ42, CSF tau, CSF phosphotau 181, hippocampal volume, entorhinal cortex thickness, MMSE, CDR, ADAS-cog, NPI, glucose, or insulin metabolism The authors advise caution when interpreting the findings related to the altered biomarker trajectories, suggesting that further analysis and consideration are necessary. However, a pilot study conducted on individuals with mild cognitive decline in AD demonstrated positive outcomes in terms of cognitive function and brain metabolism. Additional studies suggest that RV may have the potential to seal a leaky BBB and contribute to cognitive and functional improvement in AD patients. In a proof-of-concept study, RV was found to reduce glycated hemoglobin (HbA1c), preserve hippocampal volume, and enhance hippocampal resting-state functional connectivity in individuals at risk for dementia [56].

Another systematic review reported that compared to the group receiving a placebo, the administration of RV demonstrated a mitigating effect on the decline of Mini-Mental State Examination (MMSE) scores, Alzheimer’s Disease Cooperative Study—Activities of Daily Living (ADCS-ADL) scores, and CSF Aβ42 levels over the course of the 52-week trial. However, there was no observed alteration in tau levels. Furthermore, RV and its primary metabolites were detectable in both plasma and CSF. The decline in CSF Aβ40 and plasma Aβ40 levels was more pronounced in the placebo group compared to the RV treatment group, resulting in a significant difference at week 52. This means that the levels of CSF Aβ40 and plasma Aβ40 in the placebo group decreased more significantly over time compared to the RV treatment group. Notably, there was a significant decrease in the mean values of the RV group when compared to the placebo group (*p* < 0.03) [60].

In addition, Kocatürk et al. 2022 found that administering 500 mg of RV for a duration of one year resulted in reductions in CSF Aβ40 and CSF Aβ42 levels, as well as a decrease in serum Aβ40 levels in individuals with AD [58]. Likewise, Xu Lou et al., 2023 reported that RV intake, irrespective of dosage, can enhance brain volume, reduce MMSE scores, and improve AD scores in AD patients. In individuals with mild cognitive impairment, RV prevents the decline in Standard Volumes of Interest and increases Resting-state Functional Connectivity scores. Moreover, the group receiving RV exhibited notable improvements, reflected in higher rates of improvement indicated by good rate, MMSE scores, and FIM scores (*p* < 0.05), as well as lower clinical indicators and ADAS-cog scores (*p* < 0.001). Additionally, trans-RV has demonstrated a neuroprotective effect in patients with mild to moderate AD, as evidenced by change scores on ADAS-cog, MMSE, ADCS-ADL, and NPI showing less deterioration in the treatment group compared to the control group, although none of the change scores reached statistical significance [61].

In summary, the potential outcomes of taking RV from these human systematic reviews [56, 58, 60, 61] are as follows:

- Potential to traverse the BBB, influencing cognitive function and brain metabolism.
- Positive outcomes in cognitive function and brain metabolism in individuals with mild cognitive decline in AD.
- Mitigating effect on the decline of MMSE and ADCS-ADL scores in AD patients.
- Reduction in CSF Aβ40 and plasma Aβ40 levels, while a more pronounced decline observed in the placebo group.
- Reductions in CSF Aβ40, CSF Aβ42, and serum Aβ40 levels in individuals with AD.
- Enhancements in brain volume and resting-state functional connectivity in AD patients and individuals with mild cognitive impairment.
- Neuroprotective effect in patients with mild to moderate AD, while showing less deterioration in change scores on ADAS-cog, MMSE, ADCS-ADL, and NPI in the treatment group compared to the control group.

### Animal systematic reviews

Two systematic reviews [57, 59] reported in this regard that RV supplementation provided protective benefits against memory loss and brain pathology in AD transgenic (3xTg-AD) mice, as well as improving cognitive function in healthy nontransgenic (NoTg) mice. In addition, RV reduced anxiety levels and the accumulation of Aβ and phosphorylated tau (p-tau) aggregates specifically in the hippocampus of 3xTg-AD mice. The beneficial effects of RV were attributed to its activation of AMP-activated protein kinase (AMPK), leading to the upregulation of SIRT1 and cAMP response element-binding protein (CREB) [68]. Furthermore, the findings indicated that RV effectively reduced inflammation in both rat astrocytes and N9 microglia cell lines, suggesting that targeting the NF-κB signaling pathway could be a significant approach in the treatment of AD [69].

In addition to such findings, a long-term dietary supplementation of RV (150 mg/kg per day) significantly enhanced cognitive abilities and decreased AD markers in the brains of SAMP8 mice. These markers included reduced levels of Aβ42, decreased p-tau, increased phosphorylation of glycogen synthase kinase 3β (GSK-3β) at Ser9, and decreased expression of TNFα, IL-6, and IL-1β [70]. Further, RV effectively reduced the levels of reactive oxygen species (ROS), indicating its potential in preventing cognitive decline and neurochemical changes. Not to mentioned that RV facilitated neural repair in the mouse model of AD [71]. Of note, RV-induced mitophagy, which a process that eliminates damaged mitochondria to maintain their quality, has a protective effect against oxidative damage caused by Aβ in PC12 cells in in vitro AD model [72].

In addition to the aforementioned, an animal systematic review [57] that assessed 19 animal studies reported that RV-fed mice, irrespective of dosage, showed significant declines in Aβ counts and burden in various brain regions, including the medial cortex, striatum, and hypothalamus. Additionally, RV-induced activation of SIRT1 decreased Aβ42 and Aβ40 (*p* < 0.05) accumulation in SAMP8 animals. The study also revealed that RV effectively sustained the integrity of the BBB and inhibited Aβ1–42 from crossing it and accumulating in the hippocampus. More interestingly, the group of mice treated with RV showed a noteworthy reduction in Aβ42 levels compared to the control group that received the vehicle treatment (*p* < 0.00001) [57].

In addition to the accumulation of Aβ in the brain, abnormalities in the tau protein are also considered a major factor in the development of AD [73]. Studies show RV reduces hyperphosphorylated tau levels in AD mice by decreasing CDK5 and GSK3β activity, preventing tau phosphorylation at Ser396, and lowering p-tau levels in the cortex (*p* < 0.01) and hippocampus (*p* < 0.05). Further, RV reverses SIRT1 inactivation and tau hyperphosphorylation in both rats and mice. This suggests that active SIRT1 plays a role in attenuating tau hyperphosphorylation by reducing ERK1/2 phosphorylation (*p* < 0.05) and regulating neuronal PP2A activity. Noteworthy, RV treatment demonstrated cognitive improvement in rats with early AD by reducing tau activity and the activity of the Aβ–caspase3–Akt–GSK-3β-tau pathway (*p* < 0.05). These findings suggest that RV holds promise as a potential therapeutic approach for AD [57].

In summary, the results of RV administration from these animal systematic and meta-analysis reviews [57, 59] are as follows:

- Protective benefits against memory loss and brain pathology in 3xTg-AD.
- Improved cognitive function in healthy NoTg.
- Reduction in anxiety levels and accumulation of Aβ and p-tau aggregates in the hippocampus of 3xTg-AD mice.
- Activation of AMPK, leading to upregulation of SIRT1 and CREB.
- Reduction of inflammation in rat astrocytes and N9 microglia cell lines.
- Enhanced cognitive abilities and decreased AD markers in the brains of SAMP8 mice, including reduced levels of Aβ42 and p-tau.
- Decreased expression of TNFα, IL-6, and IL-1β in the brains of SAMP8 mice.
- Reduction of ROS.
- Facilitation of neural repair in a mouse model of AD.
- Significant declines in Aβ counts and burden in various brain regions in RV-fed mice.
- Sustained integrity of the BBB and inhibition of Aβ1–42 accumulation in the hippocampus.
- Reduction of hyperphosphorylated tau levels in AD mice.
- Reversal of SIRT1 inactivation and tau hyperphosphorylation in rats and mice.
- Cognitive improvement in rats with early AD.

Table 2 provides a summary of the findings derived from both human and animal studies investigating the effects of RV intake in cases of AD.

**Table 2:** A summary of the impact of RV on AD cases from human and animal systematic reviews analyzed in this umbrella review [56–61]

Studies	Findings	References
Human studies	Potential impact on BBB traversal, cognitive function improvement and metabolism in mild AD, and AD biomarkers reduction	[56]
	Mitigation of MMSE and ADCS-ADL decline in AD patients	[60]
	Reduction in CSF Aβ40, Aβ42, and serum Aβ40 in AD individuals	[58]
	Improving brain volume, reducing MMSE scores, significant improvements in clinical indicators and ADAS-cog scores, and showing less deterioration in ADAS-cog, ADCS-ADL, and NPI scores	[61]
Animal studies	Protective effects against memory loss, cognitive enhancement, and reduction of AD-related markers	[57, 59]
	Reduced anxiety levels, lowered Aβ and p-tau in hippocampus of AD model mice	[57, 59]
	Activation of AMPK, reduced inflammation in rat astrocytes and microglia	[57, 59, 68]
	Reduced pro-inflammatory cytokines, ROS, neural repair in AD models	[70, 71]
	Decreased hyperphosphorylated tau, SIRT1 reactivation in animal models, significant reductions in Aβ burden, and sustained BBB integrity in RV-fed mice	[57]

## Discussion

This comprehensive overview of six systematic reviews [56–61] examining the effects of RV in AD cases, both in humans and animals, reveals significant evidence supporting RV intake as a therapeutic or protective agent for individuals with AD. However, it should be noted that further studies with reduced limitations are necessary, as a few human studies have reported no significant effects of RV intake in AD protection.

Recent research suggests that RV may hold therapeutic potential for AD [33–36]. This is due to its ability to exert anti-amyloidogenic effects [74], provide neuroprotection [75], promote neurogenesis [76], and alleviate oxidative stress [77]. However, it is important to note that not all studies consistently report positive outcomes [39, 40]. Furthermore, existing systematic reviews on RV and AD face limitations, such as variations in study designs, participant characteristics, dosage regimens, treatment durations, and outcome measures. These limitations can introduce inaccuracies and potentially overlook confounding factors. Therefore, the objective of this study was to critically evaluate the evidence while addressing these limitations.

The question that arose was whether RV can have a positive impact on AD. After evaluation included studies [56–61], despite some limitations that will be discussed at the end of this section, we found that RV can show promise in various aspects of AD treatment. Indeed, all these studies mentioned that RV has protective effects against memory loss and cognitive decline, reduces AD-related markers such as Aβ and p-tau, and improves cognitive function and brain metabolism in AD cases. The forthcoming paragraphs will discuss the potential mechanisms through which RV can exert these effects.

Most studies suggest that RV has the ability to cross the BBB [78, 79], which is a protective barrier that separates the bloodstream from the brain. By traversing the BBB, RV can directly access the brain tissue and exert its effects. The presence of RV in the brain can influence cognitive function, which refers to various mental processes such as memory, attention, and problem-solving in AD cases [80]. Additionally, RV exhibits promising health advantages by stimulating AMPK within the brain [81, 82].

Research on the activation of AMPK by resveratrol has revealed a range of mechanisms, some of which appear intricate and occasionally conflicting. One of these mechanisms involves an increase in the ratio of AMP to ATP, as indicated by certain studies [83]. Additionally, other research suggests that the activation of AMPK by resveratrol relies on upstream serine/threonine kinases, such as LKB1 [81, 84], and calcium/calmodulin-dependent protein kinase kinase β (CaMKKβ) [85, 86]. Of interesting, it has been observed that RV can trigger AMPK independently of the AMP-to-ATP ratio [81]. This activation results in elevated glucose absorption [87, 88], improved mitochondrial function, [88], and the promotion of neuroprotective effects through anti-inflammatory action, enhanced autophagy, anti-oxidant activation through the Nrf2 pathway, and restoration of energy levels [89]. Therefore, RV’s ability to traverse the BBB is significant as it allows it to directly interact with the brain, potentially influencing cognitive function and brain metabolism.

Aβ peptides are a group of peptides involved in the formation of amyloid plaques in neurodegenerative diseases like AD [90]. The main types of Aβ peptides include Aβ40, Aβ42, and Aβ38, with Aβ42 being more prone to aggregation and considered more toxic [91]. Aβ43 and other variants like Aβ37, Aβ39, Aβ45, and Aβ46 also exist but are less common [92, 93]. The relative levels and aggregation properties of these peptides are believed to play a role in the development and progression of AD, though their exact mechanisms are still being studied [94].

Analyzed human systematic reviews suggest that RV can lead to a reduction in CSF Aβ40 and Aβ42 levels, as well as serum Aβ40 levels, in individuals with AD. The precise mechanisms by which RV can do so is not fully understood; yet one of which is that RV may modulate amyloid precursor protein (APP) processing and favor the non-amyloidogenic pathway, thus reducing the production of Aβ40 and Aβ42 peptides [85, 95]. APP is a protein found in cell membranes that is involved in normal brain function. However, in AD, there is a disturbance in the processing of APP, resulting in the accumulation of Aβ peptides, including Aβ40 and Aβ42, which are the hallmark of AD [96]. APP can be processed through two main pathways: The amyloidogenic pathway and the non-amyloidogenic pathway. In the amyloidogenic pathway, APP is cleaved by enzymes called β-secretases (such as β-secretase 1, or BACE1) and γ-secretases, resulting in the production of Aβ peptides [97, 98].

RV has been suggested to modulate APP processing and promote mostly the non-amyloidogenic pathway. The non-amyloidogenic pathway involves the cleavage of APP by α-secretase, which prevents the formation of Aβ peptides [99, 100]. RV has been proposed to boost the activity of α-secretase, an enzyme involved in the non-amyloidogenic cleavage of APP. When α-secretase activity is increased, it cleaves APP within the Aβ region, preventing the formation of Aβ peptides. As a result, there is a decrease in the production of Aβ40 and Aβ42, which are associated with the development of amyloid plaques in AD [101, 102]. Notably, RV can upregulate the expression of α-secretase while downregulating the expression of β-secretase. This modulation of secretase enzyme expression favors the non-amyloidogenic pathway and reduces Aβ production [103].

Of note, a study reported that RV does not affect the levels of APP holoprotein and its C-terminal proteolytic fragments, these data indicate that RV does not target an Aβ-producing activity, but rather promotes Aβ clearance [95]. Indeed, this study pointed out that RV does not hinder the production of Aβ, as it does not interfere with the enzymes responsible for generating Aβ, namely β-secretase and γ-secretase. Instead, RV facilitates the breakdown of Aβ within cells through a mechanism that involves the proteasome [95, 104]. Further research is needed to elucidate the detailed mechanisms of RV in modulating APP processing and thus reducing Aβ peptides in the context of AD.

In addition to the aforementioned, RV has been shown to upregulate SIRT1 in normal and AD cases [105, 106]. SIRT1, a sirtuin protein primarily found in neurons’ nuclei, dynamically regulates cellular processes such as aging, metabolism, and neuroprotection [107, 108]. Patients with AD and mild cognitive impairment have been found to exhibit lower levels of SIRT1 compared to healthy individuals [109], indicating that measuring serum SIRT1 levels could potentially serve as an early biomarker for AD diagnosis. In animal models of AD, the accumulation of Aβ was shown to suppress SIRT1 levels, whereas the administration of RV, a compound that activates SIRT1, significantly reduced Aβ deposition in the brain [110]. SIRT1 activation enhances the production of ADAM10, an enzyme that promotes the breakdown of APP through α-secretase activity, resulting in decreased Aβ levels in mouse brain tissue affected by AD [111]. Moreover, SIRT1 reduces the activity of ROCK1 and BACE1, leading to reduced Aβ levels [112]. For instance, in AD monkey models, interventions such as calorie restriction and SIRT1 overexpression were associated with lower brain Aβ levels, which correlated inversely with SIRT1 levels [113]. A recent study also supports the role of SIRT1 in activating α-secretase and further inhibiting Aβ production [114]. Together, these findings highlight the potential of SIRT1 as both a therapeutic target and a biomarker for AD.

**Fig. 2:**
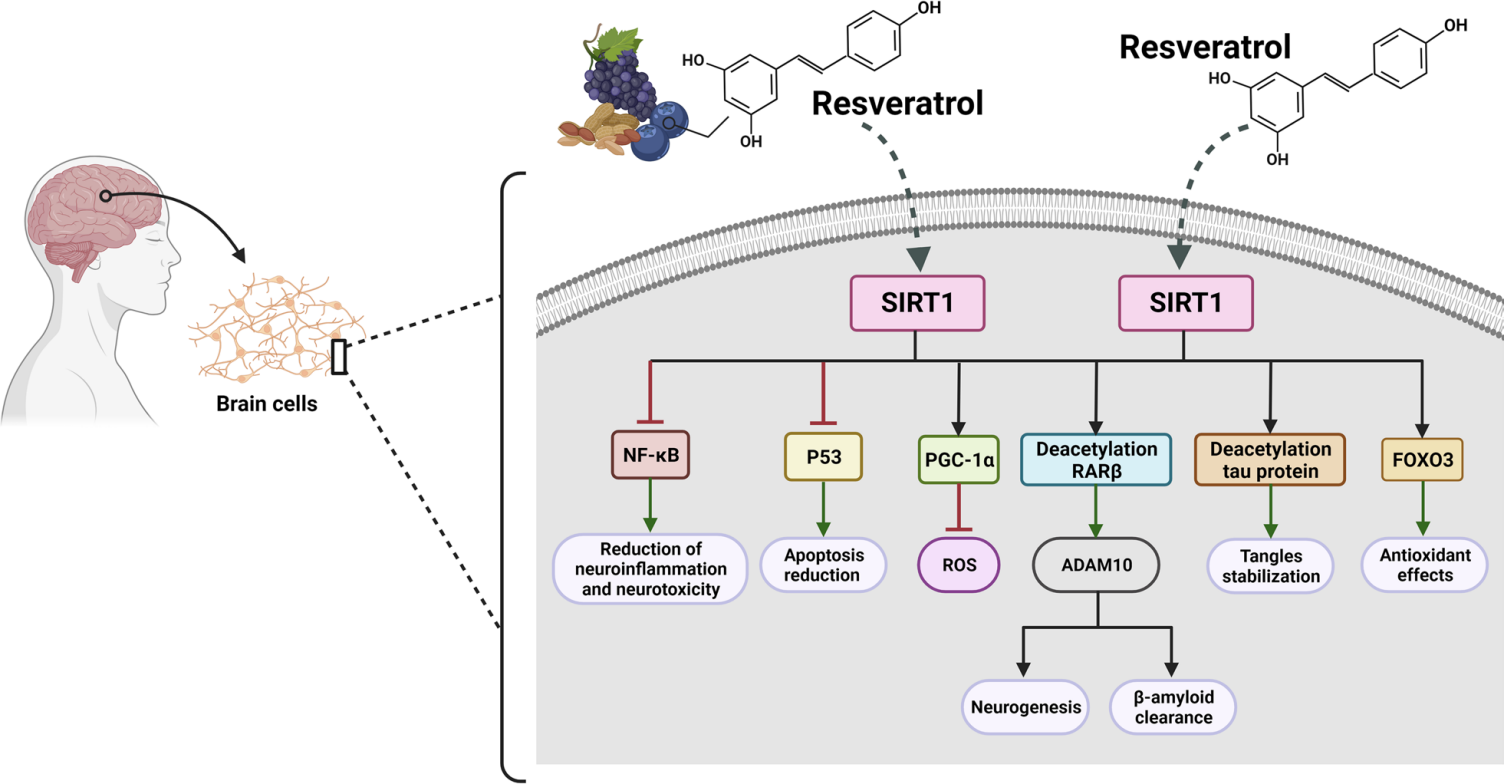
Activation of SIRT1 through RV has exhibited a range of potential benefits in brain cells. These benefits encompass neuroprotection against degeneration, increased neuronal survival, improved synaptic plasticity, anti-inflammatory properties, and enhanced blood flow and neurovascular health. RV-induced SIRT1 activation aids in reducing oxidative stress, enhancing mitochondrial function, and facilitating the clearance of toxic protein aggregates like Aβ plaques and tau tangles. Furthermore, SIRT1 plays a critical role in regulating synaptic plasticity and neurogenesis for optimal learning and memory, in particular among those who suffering from AD

In the context of AD, SIRT1 has garnered significant interest due to its involvement in pathways related to the disease’s development and progression. These pathways, along with involving Aβ metabolism, include the regulation of tau phosphorylation [115, 116], modulation of inflammation [117–119], mitigation of oxidative stress [120], and regulation of synaptic plasticity [121]. (For further gaining information about the role of SRIT1 in brain cells, see [106]) (Fig. 2).

As per these analyzed systematic reviews, it was indicated that RV has a potential to activate SIRT1; indeed, RV can interact directly with SIRT1 and stimulate its enzymatic activity related to SIRT1 [57, 59]. Upon the whole, RV has the potential to activate SIRT1 by directly interacting with the protein and enhancing its enzymatic activity, thereby providing beneficial in preventing AD.

Another probable mechanism is that RV activates CREB [122, 123], a transcription factor involved in memory formation and neuronal plasticity, potentially enhancing cognitive function in AD cases [124, 125]. RV stimulates the activation of CREB signaling pathways by regulating levels of cAMP [122]. This activation leads to the transcription and expression of genes related to synaptic plasticity, neuronal survival, and memory formation [122, 126, 127]. Increasing the expression of genes like brain-derived neurotrophic factor (BDNF) and its receptor TrkB, RV enhances synaptic plasticity and potentially improves cognitive function in AD cases [128–130]. Of note, progression of AD is accompanied by reduced levels of BDNF in the brain [131], blood [132], and CSF [133] of AD patients. Conversely, higher levels of BDNF in the blood have been correlated with improved cognitive function in individuals with AD [134].

### Limitations

This umbrella review on the role of RV in AD has several limitations that should be acknowledged. First, the included studies encompass a diverse range of study designs, such as randomized controlled trials, observational studies, and preclinical experiments. This variation introduces heterogeneity in the results, making it challenging to draw definitive conclusions. Differences in participant characteristics, RV dosage, treatment duration, and outcome measures across studies further contribute to the heterogeneity, limiting the ability to pool data and perform quantitative analyses.

Another limitation is the variability in RV dosages and formulations used across studies. Factors such as food intake and individual variations in metabolism can influence the absorption and bioavailability of RV [135–137]. This variability in RV dosages and formulations may contribute to inconsistent findings across studies and hinder the ability to determine the precise effects of RV on AD. The lack of standardized dosing protocols for RV limits the comparability and generalizability of the results.

Some studies included in this umbrella review did not fully account for potential confounding factors that could influence the effects of RV on AD. Factors such as genetic predisposition, coexisting medical conditions, and concomitant medication use may interact with RV and modify its effects [32, 138]. The heterogeneity in participant characteristics across studies may introduce confounding variables that are not adequately controlled for, thus limiting the ability to attribute observed effects solely to RV.

Notwithstanding these limitations, this umbrella review offers important insights into the current evidence regarding the involvement of RV in AD. However, it is crucial for future research to address the limitations identified in order to enhance the quality and applicability of the evidence. This will ultimately contribute to the development of RV-based interventions for the prevention and treatment of AD. As a result, researchers can improve the credibility and trustworthiness of the evidence, leading to more informed decision-making when considering the potential use of RV in managing AD.

## Conclusion

Taken together, this umbrella review of systematic reviews aimed to evaluate the potential impact of RV on AD. We found that RV may hold potential as a beneficial treatment for AD, based on evidence from both human and animal studies. One key factor that contributes to its effectiveness is RV’s ability to cross the BBB, allowing it to directly impact cognitive function and brain metabolism. RV achieves this by modulating AMPK and influencing the processing of APP. Through this modulation, RV helps reduce the accumulation of Aβ peptides and tau proteins, which are characteristic of AD. Additionally, RV has been found to increase the levels of SIRT1, CREB, and BDNF, which are proteins associated with neuronal health and protection. These combined effects make RV a promising therapeutic agent for the treatment and prevention of AD.

### Supplementary Information

**Additional file 1**. Appendix.

**Additional file 2**. PRIOR checklist.

## Data Availability

The search method, list of papers that were included and excluded, the data extraction process, and the quality evaluation are all accessible in the paper and upon reasonable request from the corresponding author.

## Abbreviations

AD: Alzheimer’s disease
Aβ: Beta-amyloid plaques
NFTs: Neurofibrillary tangles
AChE: Acetylcholinesterase
RV: Resveratrol
SIRT1: Silent Information Regulator-1
MMSE: Mini-Mental State Examination
ADCS-ADL: Alzheimer’s Disease Cooperative Study—Activities of Daily Living
p-tau: Phosphorylated Tau
AMPK: AMP-activated protein kinase
CREB: CAMP Response Element-Binding Protein
GSK-3β: Glycogen Synthase Kinase 3β
ROS: Reactive oxygen species
APP: Amyloid precursor protein
BDNF: Brain-derived Neurotrophic Factor
