# Supplementary material for: Unraveling the therapeutic efficacy of resveratrol in Alzheimer’s disease: an umbrella review of systematic evidence
Source: Nutr Metab (Lond). 2024 Mar 19;21:15. doi: 10.1186/s12986-024-00792-1 (PMC10953289; doi:10.1186/s12986-024-00792-1)
Supplement: Supplementary file 1 — Additional file 1. Appendix. [file 12986_2024_792_MOESM1_ESM.docx]

**Appendix 2**

| **Databases** | **Search Terms** |
| --- | --- |
| **Embase** | #1 resveratrol/ or wine.mp. or “SIRT1 activator”.mp. or “3,5,4'-trihydroxy-trans-stilbene”.mp. or “cis- and trans-resveratrol”.mp. or peanuts.mp. or “resveratrol supplement”.mp. |
|  | #2 “Alzheimer's disease”.mp/ or “neurodegenerative conditions”.mp/ or “cognitive function”.mp/ or “beta-amyloid”.mp/ or “neurofibrillary tangles”.mp/ or “tau protein”.mp |
|  | #3 (#1 AND #2) |
|  | #4 (#3 AND “systematic review” [Title/Abstract] OR “meta-analysis” [Title/Abstract] |
| **MEDLINE (PubMed)** | #1 (“resveratrol” [Title/Abstract] OR “wine” [Title/Abstract] OR “SIRT1 activator” [Title/Abstract] OR “3,5,4'-trihydroxy-trans-stilbene” [Title/Abstract] OR “cis- and trans-resveratrol” [Title/Abstract] OR “peanuts” [Title/Abstract] OR “resveratrol supplement” [Title/Abstract]) |
|  | #2 (“Alzheimer’s disease” [Title/Abstract] OR “neurodegenerative conditions” [Title/Abstract] OR “cognitive function” [Title/Abstract] OR “beta-amyloid” [Title/Abstract] OR “neurofibrillary tangles” [Title/Abstract] OR “tau protein” [Title/Abstract] |
|  | #3 (#1 AND #2) |
|  | #4 (#3 AND “systematic review” [Title/Abstract] OR “meta-analysis” [Title/Abstract] |
| **Cochrane Library** | #1 (“resveratrol” [Title/Abstract] OR “wine” [Title/Abstract] OR “SIRT1 activator” [Title/Abstract] OR “3,5,4'-trihydroxy-trans-stilbene” [Title/Abstract] OR “cis- and trans-resveratrol” [Title/Abstract] OR “peanuts” [Title/Abstract] OR “resveratrol supplement” [Title/Abstract]) |
|  | #2 (“Alzheimer’s disease” [Title/Abstract] OR “neurodegenerative conditions” [Title/Abstract] OR “cognitive function” [Title/Abstract] OR “beta-amyloid” [Title/Abstract] OR “neurofibrillary tangles” [Title/Abstract] OR “tau protein” [Title/Abstract] |
|  | #3 (#1 AND #2) |
|  | #4 (#3 AND “systematic review” [Title/Abstract] OR “meta-analysis” [Title/Abstract] |
| **Web of Science** | #1 TS = (“resveratrol” OR “wine” OR “SIRT1 activator” OR “3,5,4'-trihydroxy-trans-stilbene” OR “cis- and trans-resveratrol” OR “peanuts” OR “resveratrol supplement”) |
|  | #2 TS = (“Alzheimer’s disease” OR “neurodegenerative conditions” OR “cognitive function” OR “beta-amyloid” OR “neurofibrillary tangles” OR “tau protein” |
|  | #3 (#1 AND #2) |
|  | #4 (#3 AND “systematic review” [Title/Abstract] OR “meta-analysis” [Title/Abstract] |
| **Epistemonikos** | #1 (“resveratrol” [Title/Abstract] OR “wine” [Title/Abstract] OR “SIRT1 activator” [Title/Abstract] OR “3,5,4'-trihydroxy-trans-stilbene” [Title/Abstract] OR “cis- and trans-resveratrol” [Title/Abstract] OR “peanuts” [Title/Abstract] OR “resveratrol supplement” [Title/Abstract]) |
|  | #2 (“Alzheimer’s disease” [Title/Abstract] OR “neurodegenerative conditions” [Title/Abstract] OR “cognitive function” [Title/Abstract] OR “beta-amyloid” [Title/Abstract] OR “neurofibrillary tangles” [Title/Abstract] OR “tau protein” [Title/Abstract] |
|  | #3 (#1 AND #2) |
|  | #4 (#3 AND “systematic review” [Title/Abstract] OR “meta-analysis” [Title/Abstract] |
| **Google Scholar** | #1 resveratrol/ or wine.mp. or “SIRT1 activator”.mp. or “3,5,4'-trihydroxy-trans-stilbene”.mp. or “cis- and trans-resveratrol”.mp. or peanuts.mp. or “resveratrol supplement”.mp. |
|  | #2 “Alzheimer's disease”.mp/ or “neurodegenerative conditions”.mp/ or “cognitive function”.mp/ or “beta-amyloid”.mp/ or “neurofibrillary tangles”.mp/ or “tau protein”.mp. |
|  | #3 (#1 AND #2) |
|  | #4 (#3 AND “systematic review” [Title/Abstract] OR “meta-analysis” [Title/Abstract] |

**Mp**: “Multi-Purpose” or “Multiple Field”. It indicates that the specified term can appear in various fields of the database, such as title, abstract, or full text.

**TS**: “Topic Search”, which indicates that the specified search terms should be applied to relevant fields such as title, abstract, keywords, and author keywords.
